# Study the effect of *Lactobacillus plantarum* ATCC 14917 for caries prevention and anti-obesity

**DOI:** 10.3389/fnut.2024.1511660

**Published:** 2024-12-24

**Authors:** Wei Yang, Mingxin Jiang, Bairu Chen, Kongzhao Jiang, Nan Ma, Yimin Li, Meng Wang, Meihua Bao, Chengyue Wang, Xiaopeng Yang

**Affiliations:** ^1^Department of Pedodontics, Affiliated Stomatology Hospital of Jinzhou Medical University, Jinzhou, China; ^2^Collaborative Innovation Center for Health Promotion of Children and Adolescents of Jinzhou Medical University, Jinzhou, China; ^3^Department of Micro-endodontics, Affiliated Stomatology Hospital of Jinzhou Medical University, Jinzhou, China; ^4^Department of Prosthetics, Affiliated Stomatology Hospital of Jinzhou Medical University, Jinzhou, China

**Keywords:** *Lactobacillus plantarum*, *Streptococcus mutans*, dental caries, obesity, gut microbiota

## Abstract

**Introduction:**

A complicated scenario where “multiple disease threats coexist and multiple health influencing factors are intertwined” is demonstrated by the fact that dental caries, obesity myopia and scoliosis have emerged as global public health issues. The problem of diseases co-existing in living things can be resolved by using probiotics. *Lactobacillus plantarum*, has gained attention recently due to its probiotic properties, useful traits, and potential medical applications.

**Objective:**

Examining the anti-obesity and anti-caries effects of *L. plantarum* ATCC 14917 on dental caries and obese rat models caused by a high-fat and high-sugar diet is the aim of this study.

**Method:**

*In vitro*, we assessed the *L. plantarum* strain’s probiotic properties, such as its antibacterial activity and ability to build biofilms, to determine its ability to inhibit *Streptococcus mutans*. Prior to the *in vivo* experiment, the subsist test for *L. plantarum* ATCC 14917 was carried out by mimicking its capacity to lower blood sugar and blood lipid levels as well as its tolerance to gastrointestinal disorders. In order to assess the health promotion effect of *L. plantarum in vivo*. Three-week-old rats were fed a high-sugar, high-fat diet for 8 weeks. They were split into three groups: the control group (Control), the caries and obesity group (CA _OB) and the caries and obesity treated with *L. plantarum* ATCC14917 group (LP). *L. plantarum* ATCC 14917 was applied during the experiment, and the associated indices were then thoroughly assessed. These included the use of Mirco-CT to calculate the enamel volume, the staining of liver and fat cell sections, serological analysis, and 16S rRNA sequencing of feces.

**Results:**

It was proved that the *L. plantarum* could inhibit the proliferation of *S. mutans* and remove dental plaque biofilm in time, which showed the remarkable effects of anti-caries *in vitro*. The demineralization rate of enamel decreased by 44.10% due to the inhibition of acid production by pathogenic bacteria. Moreover, In intestinal and stomach juice simulations, *L. plantarum* has a high survival rate. The characteristics of bacterial activity in a wide range of pH could degrade triglycerides and glucose *in vitro* smoothly. The LP group demonstrated it by reducing animal weight, serum biochemical indices, and HE-stained adipocytes as compared to the CA_OB group. 16S rRNA sequencing data showed that a high-fat and high-sugar diet induced the imbalance of intestinal flora, which showed an increase in microbial abundance, including *unclassified_o__Clostridia_UCG-014*, *unclassified_f__Oscillospiraceae*, *Turicibacter*, *unclassified_f__Lachnospiraceae*, *Clostridium_sensu_stricto_1*. After the intervention of *L. plantarum*, the number of *Lactobacillus*, *Limosilactobacillus*, *unclassified_f__Muribaculaceae*, *Blautia*, *Faecalibaculum* increased significantly.

**Conclusion:**

Therefore, *L. plantarum* ATCC 14917 performed the potential of reducing tooth decay and controlling weight gain by a single strain. Support the management of dental caries and obesity, and establish a foundation for future functional food research and development.

## Graphical abstract

Overview of *Lactobacillus plantarum* ATCC 14917 for caries prevention and weight loss *in vivo* and *in vitro*.
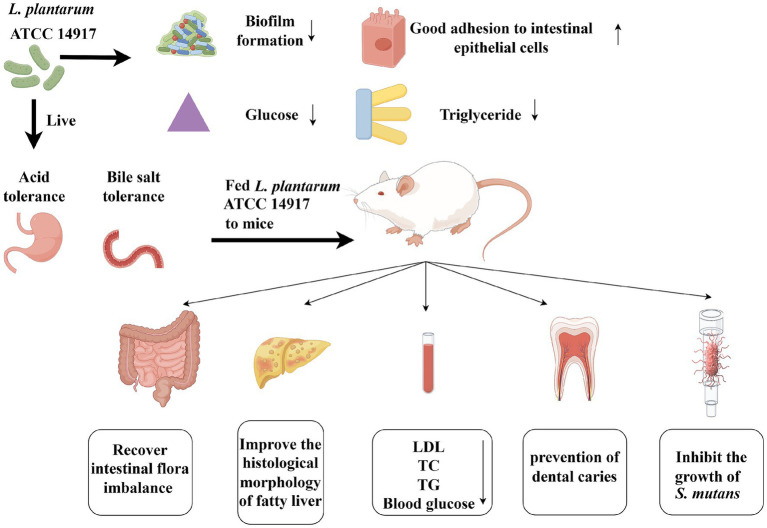

## Highlights

- *L. plantarum* ATCC 14917 could treat dental caries and obesity at the same time.
- *L. plantarum* ATCC 14917 degraded glucose and triglycerides well *in vitro.*
- *L. plantarum* ATCC 14917 recovered the intestinal flora imbalance.

## 1 Introduction

Dental caries and obesity are considered to be chronic, highly prevalent and multifactorial diseases, which are harmful to the life and health of children and adolescents (1–3). *Streptococcus mutans* is the main pathogenic bacteria of dental caries. Because of its excellent ability to make full use of sucrose to synthesize extracellular glucan and intracellular polysaccharide, which can promote adhesion to form dental plaque biofilm and provide sufficient energy for itself (4). Moreover, acidification of the microenvironment in biofilm leads to demineralization of enamel and decay (5). Obesity is caused by a combination of biology and environment variables, which are characterized by excessive adipose tissue, dysfunction and changes of intestinal microbiota (6, 7).

Recent years have seen a lot of emphasis focused on the connection between dental caries and obesity, and various studies have examined this relationship (8, 9). Older children and adolescents who are obese have a higher risk of developing dental caries, and there is a significant positive correlation between dental caries and weight gain (10–12). Reasonable mechanisms have been put out for the increasing prevalence and or severity of caries in overweight or obesity individuals. One explanation is that poor eating habits and excessive carbohydrate intake are the primary causes (13–15). Additionally, because obesity is a chronic inflammatory illness, decreased salivary flow can exacerbate oral discomfort (16). As a result, obese children may be more susceptible to dental caries (17, 18). Obesity and dental cavities are associated with parents’ poor income and educational attainment (19). Children who are overweight or obese have a higher risk of dental cavities (20, 21). As a result, the association between dental caries and obesity is assessed across a range of social and environmental contexts, as well as across cultural, dietary, and obesity-causing behaviors.

Excessive carbohydrate intake will not only promote the occurrence of dental caries, but also accumulate fat (22). Recent studies have unveiled the existence of the oral-intestinal axis, highlighting significant relevance among specific genes, their metabolites, and the development of dental caries and obesity, which may be the potential target of intervention (23–25). The chronic usage of antibiotics has been shown to disrupt the delicate equilibrium of both oral and intestinal ecosystems, potentially leading to an increase in bacterial resistance (26). Consequently, there is a pressing need for the development and implementation of safer and more effective preventive strategies for caries and obesity.

Probiotic therapy emerges as a promising solution, as it can help restore the balance of oral microbiota, thereby preventing and treating a range of oral health issues, including caries and periodontology (27). Among these probiotics, *Lactobacillus plantarum* stands out as a facultative heterotrophic fermentation organism with unique regulatory properties (28). It has been found to address disturbances in glucose and lipid metabolism that may arise from diets high in fats and sugars (29). Its beneficial effects may involve different mechanisms, including improving blood glucose and blood lipid metabolism, regulating intestinal microbiota (30).

By using the agar diffusion method and crystal violet staining method, *L. plantarum* of antibacterial activity and antibacterial membrane capacity against *S. mutans* were detected and its aggregation activity was investigated. We evaluated the strain’s probiotic characteristics, including *L. plantarum* for tolerance in the gastrointestinal tract and its ability to consume fat and sugar. In obese rats with dental caries caused by a high-sugar and high-fat diet, the gut microbial composition and demineralized enamel volume were observed using 16S rRNA and Micro-CT. This research of purpose was to assess the inhibitory effect of *L. plantarum* on dental caries and obesity. It provide a fresh perspective on the use of probiotics to manage illnesses, and support as a foundation for the development of functional foods.

## 2 Materials and methods

### 2.1 Bacterium and supernatant preparation

*Lactobacillus plantarum* (*L. plantarum* ATCC 14917, CICC, China) and *S. mutans* (*S. mutans* ATCC 25175, CGMCC, China) were grown in DeMan-Rogosa-Sharpe (MRS) medium (Hopebio., Ltd., Qingdao, China) and Brain-Heart-Infusion (BHI) medium (Hopebio., Ltd., Qingdao, China) respectively. All strains were fostered at 37°C for 16–24 h, and were adjusted to OD_600_ = 1, respectively. The bacterial culture was centrifuged and filtered to extract the supernatant of *L. plantarum*.

### 2.2 Antimicrobial property of *Lactobacillus in vitro*

#### 2.2.1 *Lactobacillus* bacteriostatic test

*Lactobacillus plantarum* culture solution was added to an Oxford cup and the growth inhibition diameter was measured after 24 h of culture with MRS liquid culture medium as control. Absorption method was partially modified to test antibacterial activity of *L. plantarum*. *L. plantarum* supernate an MRS medium were mixed into 100 μL of *S. mutans* culture solution, respectively. When the inhibition ratio is less than two after 18 to 24 h of culture, the sample demonstrated good antibacterial activity. The bacteriostatic value was obtained by Equation 1:


(1) $$A=\mathrm{lg}C_{1}-\mathrm{lg}T_{1}$$

where A represented the bacteriostatic value, C_1_ the control group’s average colony forming units (CFU), and T_1_ the experimental group’s average CFU.

#### 2.2.2 *Streptococcus mutans* in supernatant of *Lactobacillus plantarum*

Supernatant of *Lactobacillus* was to handle *S. mutans*. A final concentration of 12.5 to 100% (v/v) was employed, with MRS liquid medium serving as the control. Every 4 hours, 100 microliters of various concentrations of culture fluid were collected and put in a 96-well plate to monitor the growth conditions. The ELISA Reader was used to measure the OD value at 600 nm. An ELISA reader was used to measure optical density at 600 nm.

#### 2.2.3 Formation and removal of biofilm

To evaluate the biofilm formation and removal, crystal violet staining was employed, utilizing various concentrations of the supernatant ranging from 0 to 100% (v/v), with MRS liquid medium serving as the control. Following a 24 h culture period, it was washed with PBS for 3 times and a 15 min methanol fixation. 0.1% crystal violet was utilized for staining, followed by the dissolution of 33% glacial acetic acid. The optical densities were again measured at 600 nm to assess the effectiveness of the different concentrations on biofilm dynamics.

By adding 50% (v/v) *L. plantarum* supernatant with the same quantity at various time intervals (0 h, 6 h, 12 h, and 24 h), which was used to observe the production and removal of biofilm. MRS medium was used as the control (12 h and 24 h). Staining and determination steps are the same as above, and the biofilm reduction rate is obtained by Equation 2:


(2) $$\mathrm{Biofilm reduction rate}\;\left(\%\right)=\frac{\Delta {OD}_{\left(\begin{array}{l} \mathrm{control}\;\mathrm{group}-\\ \mathrm{treatment}\;\mathrm{group} \end{array}\right)}}{\Delta {OD}_{\left(\mathrm{control}\;\mathrm{group}\right)}}\times 100$$

Once a biofilm had formed after 24 h of culture, a dyeing working solution was made by diluting PI and N_0_ fluorescent dye with NaCl solution (Shanghai Beibo Biotechnology Co., Ltd.). After gently rinsing the side wall three times with NaCl and draining, add 200 μL of the dyeing working solution and let it sit in the dark for fifteen minutes. After washing the bacteria once with NaCl solution, resuspend them by adding the proper amount of NaCl solution. The red signal is received through the red channel, and the green signal is received through the green channel. Look through a fluorescent microscope and snap pictures.

#### 2.2.4 Auto-aggregation and co-aggregation

Specific modifications to the previously established methods were applied. Adjust the concentration of *L. plantarum* solution to OD_600_ = 0.6 ± 0.05, and the concentration of *S. mutans* solution to OD_600_ = 0.5 ± 0.05, and the bacteria separately and in a mixed manner. The absorbance of the upper liquid were measured at various time intervals. The self-polymerization and co-polymerization ability of strains at different times were judged, respectively. The Auto-aggregation ability was obtained by Equation 3:


(3) $$\mathrm{Auto}-\mathrm{aggregation}\;\left(\%\right)=\left[1–\frac{A_{t}}{A_{0}}\right]\times 100$$

A_0_ refers to the absorbance measured at 0 h, while A_t_ represents absorbance values taken at 2, 4, 6, and 24 h.

The Co-aggregation ability was obtained by Equation 4:


(4) $$Co-\mathrm{aggregation}\;\left(\%\right)=\frac{\left(\;A_{x}+A_{y}\right)\;–2A_{\mathrm{mix}}}{A_{x}+A_{y}}\times 100$$

Where A_x_ is the absorbance of 0 h *L. plantarum*, A_y_ is the absorbance of 0 h *S. mutans*, A_mix_ is the absorbance of the mixture at the 2, 4, 6, and 24 h time points.

### 2.3 Evaluation of digestive environment tolerance

#### 2.3.1 Tolerance to acid and bile salt

*Lactobacillus plantarum* was inoculated into MRS medium with pH of 3.0 and 0.3% (w/v) bile salt (Beijing Solarbio Science & Technology Co., Ltd.), respectively. The MRS culture medium served as the control. Following incubation for 3 and 24 h, the results were analyzed by evaluating the optical density at a wavelength of 600 nm. The survival rate was obtained by Equation 5:


(5) $$\mathrm{Survival rate}\left(\%\right)=\frac{\Delta {OD}_{\left(\mathrm{treatment group}\right)}}{\Delta {OD}_{\left(\mathrm{control group}\right)}}\times 100$$

#### 2.3.2 Tolerance of gastric juice and intestinal juice

*Lactobacillus plantarum* suspension was inoculated into artificial gastric juice (Shanghai yuanye Bio-Technology Co., Ltd.) and artificial intestine juice, respectively. The survival rate was obtained by Equation 5.

### 2.4 Oral and intestinal correlation

#### 2.4.1 The capability of *Lactobacillus* to scavenge glucose

*Lactobacillus plantarum* and *S. mutans* were inoculated in MRS and BHI broth containing 10% glucose. After being diluted to the same concentration (OD_600_ = 1). The glucose content after 24 h was measured by the glucose kit method (Nanjing Jiancheng Bioengineering Company, China), and the solution without bacterium was used as control. The content rate of glucose was obtained from Equation 6:


(6) $$\mathrm{Content ratio}\;\left(\%\right)=\frac{\Delta {OD}_{\left(\mathrm{treatment}\;\mathrm{group}\right)}}{\Delta {OD}_{\left(\mathrm{control}\;\mathrm{group}\right)}}\times 100$$

#### 2.4.2 The ability of *Lactobacillus plantarum* to scavenge triglycerides

A 0.5% (v/v) inoculum of both *L. plantarum* and *S. mutans* cultures were introduced into Triglyceride-MRS and Triglyceride-BHI media, incubated at 37°C for 24 h. The triglyceride content was measured utilizing the triglyceride kit method, both with and without bacteria for comparative analysisthe triglyceride content ratio is calculated according to Equation 6.

### 2.5 *In vivo* effects of *Lactobacillus plantarum*

#### 2.5.1 Animal model establishment

All procedures were conducted at the Animal Experimental Center of Jinzhou Medical University, with the animal studies receiving prior examination and approval from the Animal Ethics Committee of the same institution. The approval agreement number of animal program is 240,139. Male Sprague Dawley rats of 3-week-old were purchased and housed under controlled temperature (20°C ± 5°C) and Temperature (50% ± 10%) conditions. Rats were randomly allocated into three groups, each containing three rats. The control group (Control) was given conventional feed and sterile water, while the caries and obesity group (CA_OB) and caries and obesity treated with *L. plantarum* ATCC14917 group (LP) were given a high-fat and high-sugar feed and 5% sucrose water. The 56-day experiment included a 0–3 day antibiotic interference period during which ampicillin (0.5 μg/mL) inhibited the oral flora of the rats. The colonization time of *S. mutans* is 4–8 days. Before each use, a bacterial solution (10^8^ CFU/mL) was made using BHI culture medium. Each rat was anesthetized by inhalation. Using sterile cotton swabs, dip the *S. mutans* suspension until it is saturated. Then, in turn, daub in the respective group’s teeth, tongue, and oral mucosa, remaining at each location for 15 s. Using a blunt syringe, rinse the rat’s mouth, making sure the solution reaches every area of the mouth and plays its full part. The experimental solution was 1 mL/tube. Rats handled once daily in the morning and evening. The LP group was given 2 × 10^8^ CFU/mL *L. plantarum* bacterial solution orally from day 14 to day 56. Until the completion of the trial, *L. plantarum* was administered four times per week. To ensure microbial colonization, refrain from eating or drinking for 30 min prior to and following the inoculation. The same experimenter always performed the procedure. Every week, the rats’ weight was recorded. The animals were put down at the conclusion of the experiment, and samples were gathered.

#### 2.5.2 Micro-CT observation

Mandibular specimens were scanned by Micro-CT [PINGSENG Healthcare (Kunshan) Inc.]. The enamel was separated from the mandible with a fixed threshold and the volume of enamel was calculated to evaluate the dental caries. The demineralization rate of enamel was obtained from Equation 7:


(7) $$\mathrm{Demineralization}\;\mathrm{rate}\;\mathrm{of}\;\mathrm{enamel}\left(\%\right)=\frac{V_{t}-V_{0}}{V_{m}-V_{0}}\times 100$$

Where V_0_ was the volume of enamel in control group, V_t_ was the volume of enamel in LP group, and V_m_ was the volume of enamel in CA_OB group.

#### 2.5.3 Analysis of serum biochemistry

Serum was extracted by centrifuging blood samples at 5000 × g for 10 min after the animals were put under anesthesia. The serum was stored at-80°C until further analysis.

Utilizing the assay kits, levels of Total triglyceride (TG), Total cholesterol (T-CHO), High-density lipoprotein-cholesterol (HDL), Low-density lipoprotein-cholesterol (LDL), and Blood glucose were evaluated (Nanjing Jiancheng Bioengineering Company, China).

#### 2.5.4 Analysis of histology and staining

Hematoxylin and eosin (H&E) staining was performed on the rat liver and epididymis adipose tissues. Rat liver and epididymis fatty tissue was cleaned with regular saline, fixed for 24 h in a 10% paraformaldehyde solution, and the excess fixative was rinsed off with tap water. Following ethanol gradient dehydration, the tissue was embedded in paraffin and cut using a microtome into slices that were 4–5 microns thick. Following hematoxylin–eosin staining, followed by photography to document the findings.

#### 2.5.5 16S rRNA gene sequencing of intestinal flora

Fresh feces from each rat were collected on the 57th day under aseptic conditions on the ultra-clean workstation, put in a 5/mL freezing tube, kept in the refrigerated at-80°C, and then delivered to the firm for testing. The FastPure Stool DNA Isolation Kit (MJYH, shanghai, China) was used to extract the total DNA. The hypervariable region V3–V4 of the bacterial 16S rRNA gene were amplified with primer pairs 338F (5’-ACTCCTACGGGAGGCAGCAG-3′) and 806R (5’-GGACTACHVGGGTWTCTAAT-3′). Identification, purification and quantification of PCR products were carried out. The sequencing was based on the platform of Shanghai Maggie Biomedical Technology Co., Ltd.

#### 2.5.6 Statistical analysis

The statistical program GraphPad Prism 8.0.2 was utilized for the mapping and analysis. To analyze the differences among the groups, a one-way analysis of variance was utilized, with a significance level set at *p* < 0.05 to indicate noteworthy differences. Data were expressed as mean ± standard deviation, and each experimental condition was replicated three times to ensure reliability and consistency in the results obtained.

## 3 Results

### 3.1 Function of *Lactobacillus plantarum in vitro*

#### 3.1.1 Antibacterial ability

**Figure 1:**
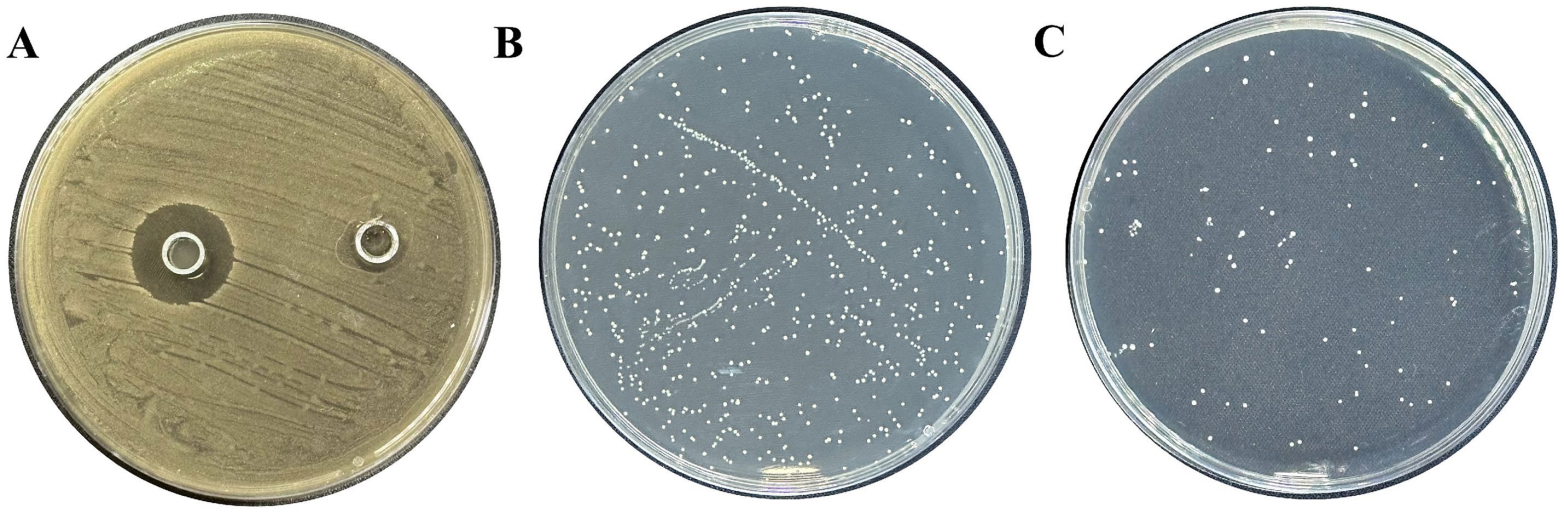
Inhibition of *L. plantarum* on *S. mutans.* **(A)** Inhibitory zone of *L. plantarum* on *S. mutans*, **(B)** CFU of *S. mutans* by MRS, **(C)** CFU of *S. mutans* by *L. plantarum* supernate. Data are represented as means ± standard deviation (SD). There were three biological duplicates (*n* = 3).

*Lactobacillus plantarum* had a substantial inhibitory effect on *S. mutans* growth, and it measured 18.85 ± 1.05 mm in diameter (Figure 1A). *Lactobacillus* exhibited a 2.24 antibiosis activity to *S. mutans*. The aforementioned findings demonstrated that *Lactobacillus* had a potent antibacterial impact (Figures 1B,C).

#### 3.1.2 Determination of bacteriostatic curve

**Figure 2:**
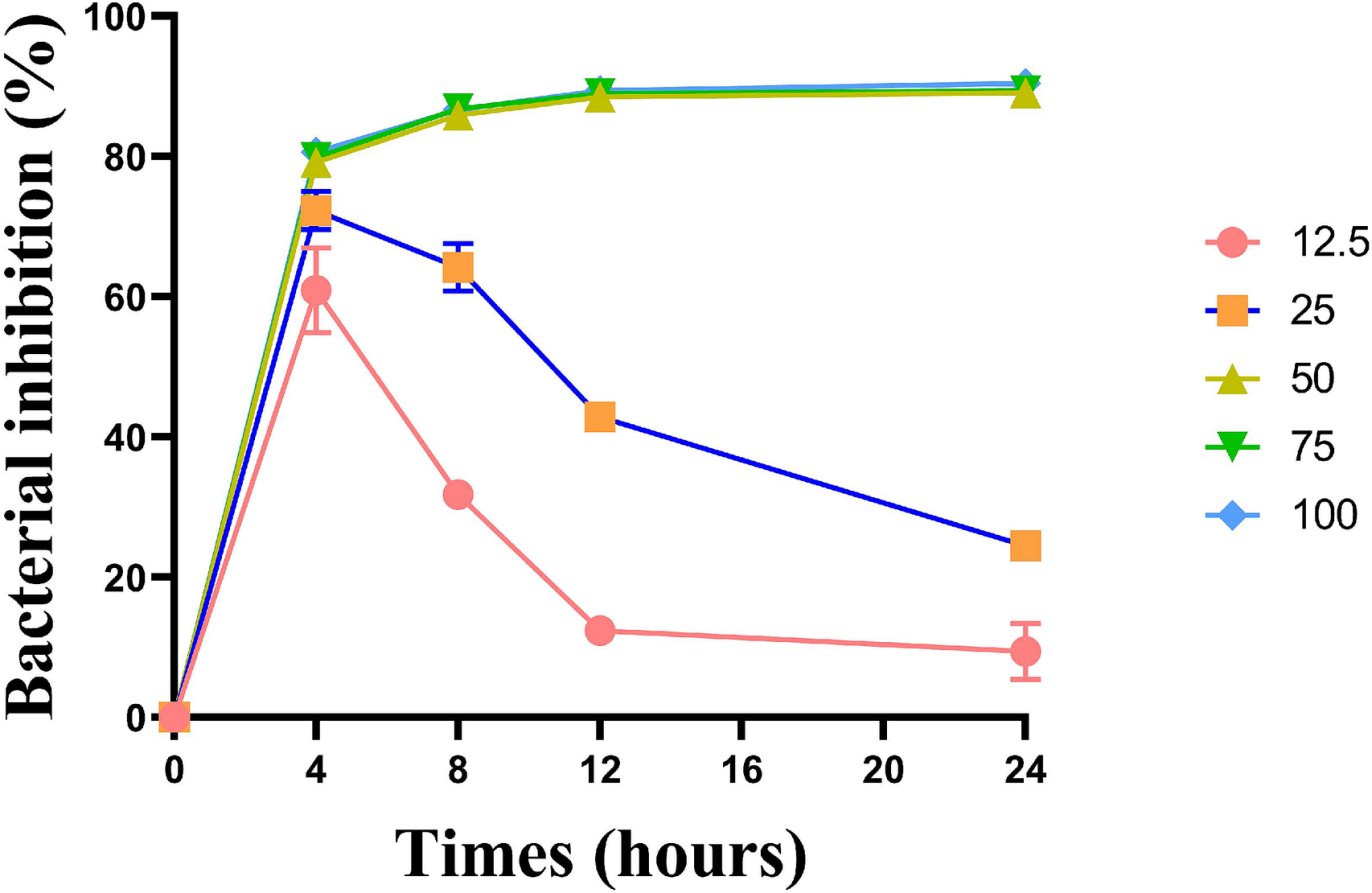
Inhibitory effect of *L. plantarum* supernate with concentration ranging from 12.5 to 100% (v/v) on the growth curve of *S. mutans*. Values are means ± SD. There were three biological duplicates (*n* = 3).

The bacteriostatic effects of the five groups of *L. plantarum* supernate started to take effect at 4 h, with the corresponding bacteriostatic rates being 60.90, 72.30, 79.18, 79.83, and 80.62%. The bacteriostatic efficacy of *L. plantarum* supernatant at concentrations of 12.5 and 25% declined with time, from 8 to 24 h (*p* < 0.05). Nonetheless, *L. plantarum* supernate still plays a good bacteriostatic role at concentrations of 50 to 100% (v/v), and the bacteriostatic rate was higher than 79% (*p* > 0.05) (Figure 2).

#### 3.1.3 Proportional distribution of crystal violet biofilm and live/dead fluorescent staining

**Figure 3:**
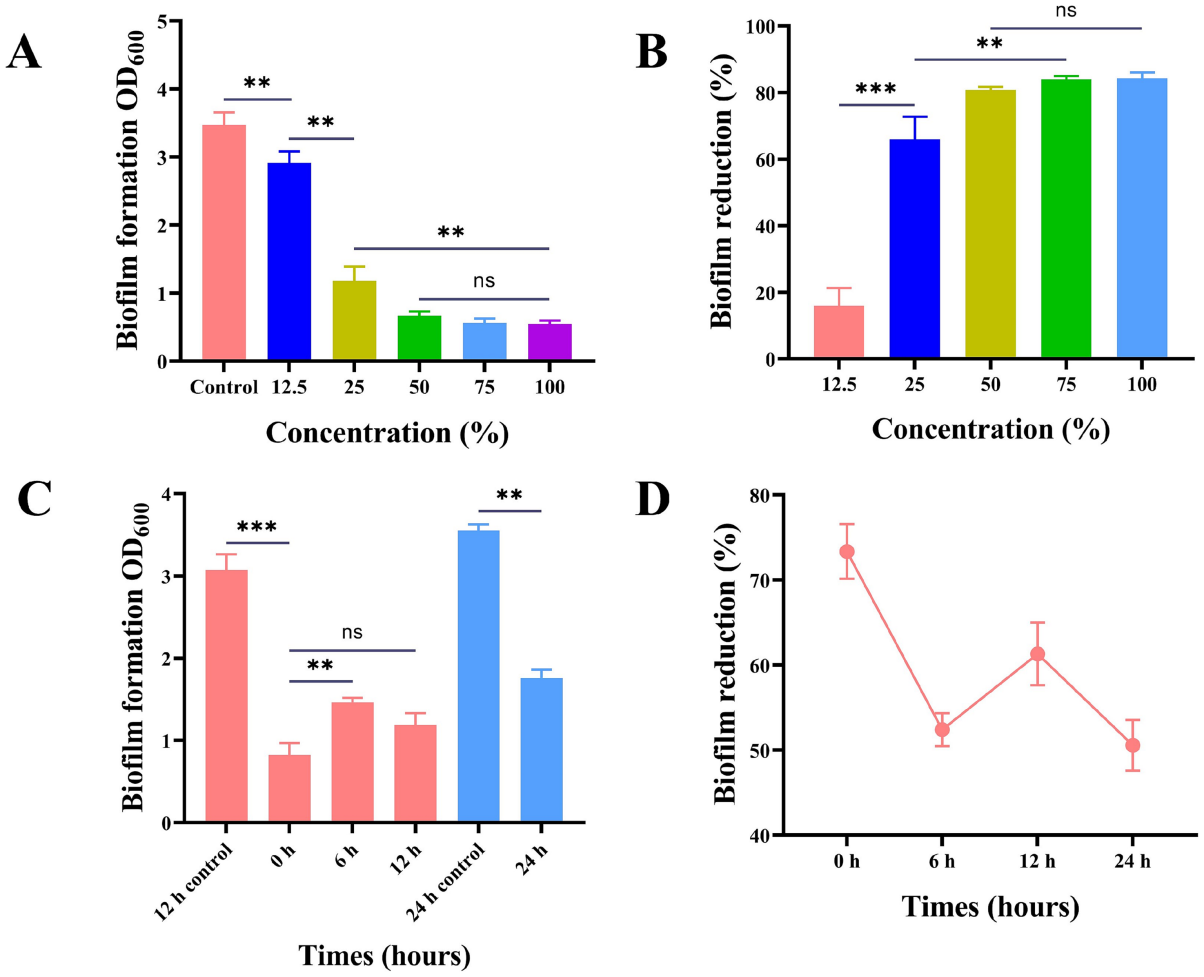
Effect of supernatant of *L. plantarum* on biofilm of *S. mutans*. **(A)** The anti-S*. mutans* biofilm effect of the *L. plantarum* supernate with the concentration ranging from 12.5 to 100% (v/v), **(B)** the reduction rate of biofilm of *S. mutans*, **(C)** the effect of adding *L. plantarum* supernate at different time points on the biomass of *S. mutans*, **(D)** Reduction rate of biofilm of *S. mutans* at various moments in time. Values are means ± SD. There were three biological duplicates (*n* = 3). * *p* < 0.05, ** *p* < 0.01, *** *p* < 0.001.

Studies have proved the influence of *Lactobacillus* on the biofilm of *S. mutans* within 24 h. *L. plantarum* supernate (0, 12.5, 25, 50, 75, 100%) exhibited varying degrees of inhibitory effects on *S. mutans* biofilm. The corresponding inhibitory rates were 15.91, 66, 80.79, and 83.99% (*p* > 0.05). The biofilm formation process is characterized by four crucial time points: the initial bacterial adhesion occurring at 0 h, initial bacterial colonization at 6 h, early biofilm development at 12 h, and the maturation of the biofilm at 24 h (Figure 3).

**Figure 4:**
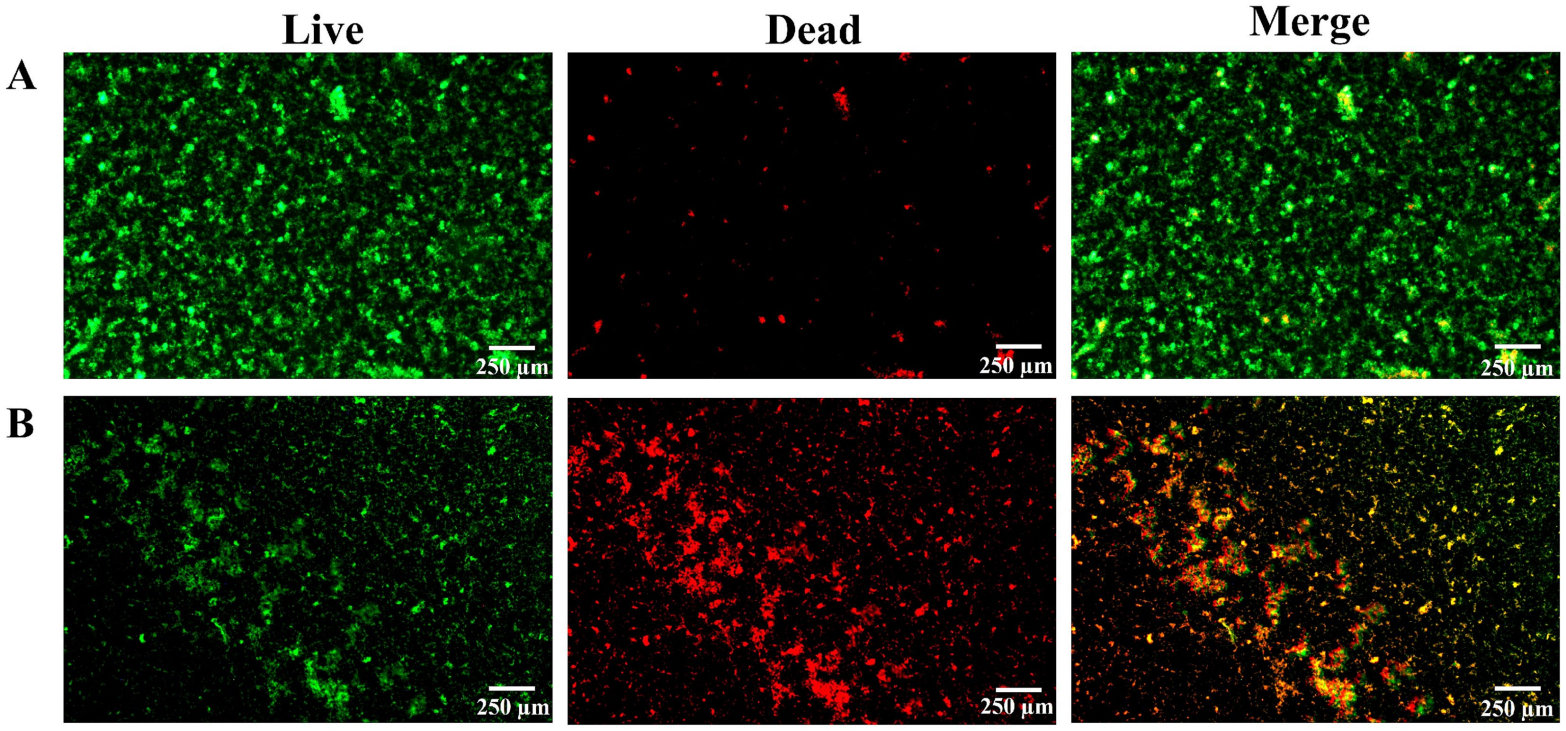
After 24 h of culture, observe the proportion of live and dead bacteria in biofilm by fluorescence staining for 30 min. **(A)** The control group, **(B)** the treatment group. Note: green was the live bacteria, red was the dead bacteria, and the superimposed color of the dead bacteria and the live bacteria was yellow or orange. There were three biological duplicates (*n* = 3).

In addition, with the intervention of *L. plantarum*, fluorescent staining was also used to measure the biofilm activity of *S. mutans*. After 24 h of culture, it was found that the colonies in the control group covered the whole field of vision, and there was no gap in the middle. The membrane was a dense network structure, mainly with green fluorescence and scattered with red fluorescence (Figure 4A). Live bacteria decreased gradually, the block structure decreased, the biofilm became sparse gradually, the red part increased, and the activity of the biofilm was obviously inhibited (Figure 4B).

#### 3.1.4 Auto-aggregation and co-aggregation

**Figure 5:**
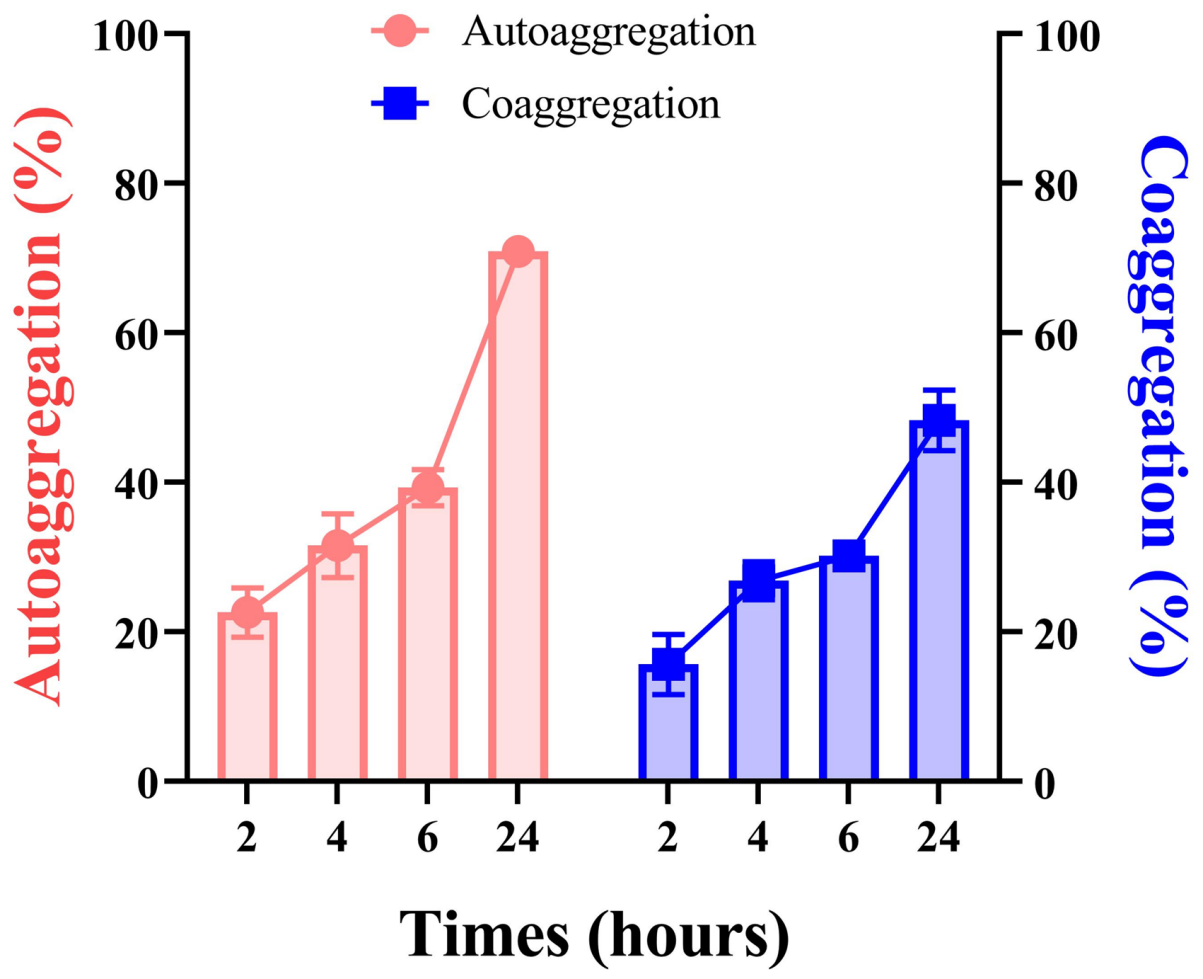
The auto-aggregation and co-aggregation ability of *L. plantarum* at 2 h, 4 h, 6 h, and 24 h. Data are represented as means ± SD. There were three biological duplicates (*n* = 3).

With time, *L. plantarum* ATCC 14917’s capacity for self-aggregation grew. The auto-aggregation rates of the strain were observed over various time intervals: 2 h, 4 h, 6 h, and 24 h, yielding rates of 22.62, 31.56, 39.32, and 70.91%, respectively. At 2 hours, the co-aggregation ability was minimal; by 4 hours, it had clearly increased. The strain under test started to agglutinate with *S. mutans*, according to the data, and after 24 h, the rate of cross-agglutination had reached 48.32% (Figure 5).

#### 3.1.5 Tolerance of the digestive environment

The properties of probiotics were evaluated using the average intestinal bile salt content, which was 0.3% (w/v). Under conditions of low pH, the survival rate of *L. plantarum* decreased significantly, falling from 73.20 to 15.18%. For the duration ranging from three to twenty-four hours in a medium containing 0.3% bile salts, the survival rate remained consistently near 80% (Table 1).

**Table 1:** Survival rate of *L. plantarum* in low pH and bile salt environment.

Time (h)	Acid resistance (%)	Bile salt resistance (%)
3	73.20 ± 6.45	87.63 ± 3.05
24	15.18 ± 0.22	82.11 ± 5.77

The number of viable bacteria decreased by 0.68 and 0.3 after 3 hours, respectively. Lactic acid bacteria have a strong ability to adapt to the tough environment of the gastrointestinal tract, as evidenced by the fact that even while their development was limited, a constant survival rate could still be ensured (Table 2).

**Table 2:** Tolerance of *L. plantarum* to simulated artificial fluid and gastrointestinal fluid.

	Initial Log (CFU/mL)	Termination Log (CFU/mL)	Survival (%)
Gastric juice	7.33 ± 0.02	6.66 ± 0.04	90.77 ± 0.51
Intestinal juice	7.37 ± 0.02	7.06 ± 0.05	95.92 ± 0.73

#### 3.1.6 Determination of triglyceride and glucose

**Figure 6:**
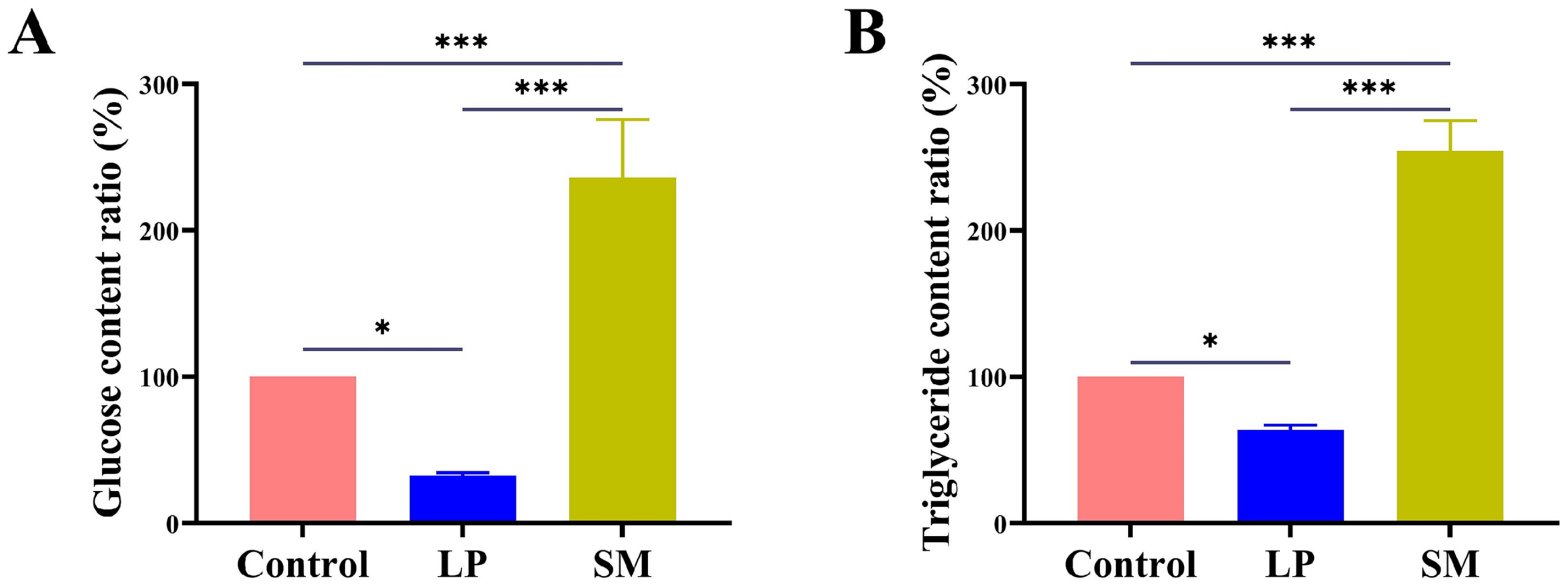
**(A)** Effects of *L. plantarum* and *S. mutans* on glucose, **(B)** effects of *L. plantarm* and *S. mutans* on triglyceride. Values are means ± SD. There were three biological duplicates (*n* = 3). * *p* < 0.05, ** *p* < 0.01, *** *p* < 0.001.

Bacteria require glucose as their primary growth substrate, and different strains exhibit diverse abilities to metabolize it. The amount of glucose content was found to be 32.54 and 236.13% in the culture medium, and the rate of lowering blood sugar and the increasing blood sugar are 67.46 and 136.13%, respectively (Figure 6A). On the other hand, the triglyceride content in the Triglyceride-MRS and Triglyceride-BHI media was 63.84 and 254.50%, respectively, and the lipid-lowering and fat-increasing rates were 36.16 and 154.50% (Figure 6B). The data above demonstrated the strain’s strong probiotic qualities.

#### 3.1.7 Micro-CT observation

**Figure 7:**
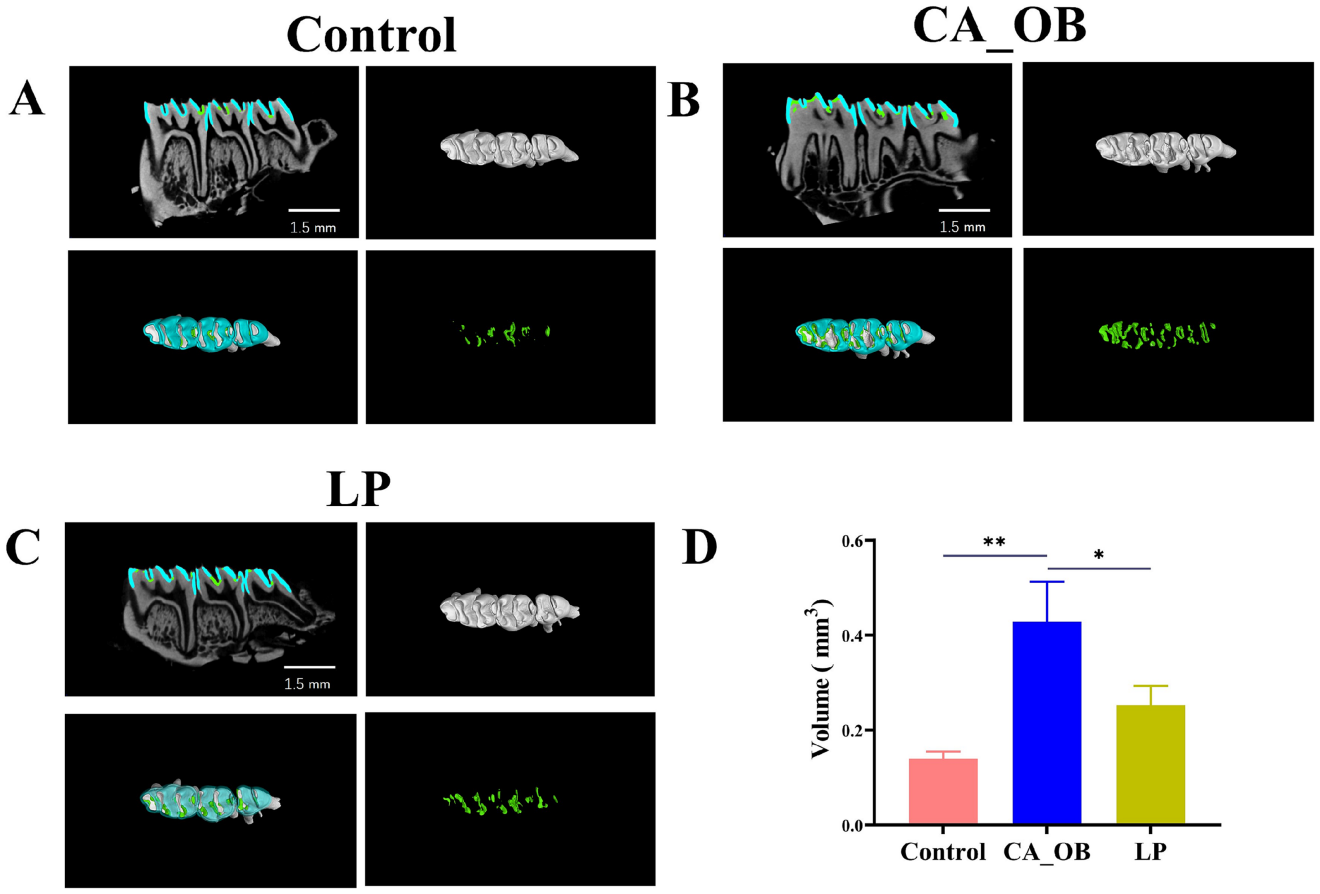
To observe the development of dental caries in rats. **(A–C)** The left maxillary tooth’s 3D microcomputer tomography pictures show demineralized enamel (cyan), two-dimensional axial and demineralized enamel (green), **(D)** demineralized enamel. Data are represented as means ± SD. There were three biological duplicates (*n* = 3). * *p* < 0.05, ** *p* < 0.01, *** *p* < 0.001.

Rat molar enamel volumes were computed for every experimental group. The model demonstrating the establishment of caries is further validated by the considerable amount of demineralized enamel found in the CA_OB group when compared to the Control group. The volume of demineralized enamel in LP group is similar to that in Control group. In addition, the demineralization rate of enamel of the LP group is similar to that of the Control group. The above results indicate that *L. plantarum* has an obvious inhibitory effect on dental caries in animal models (Figure 7).

#### 3.1.8 Assessment of body weight and serum indices in rats

**Figure 8:**
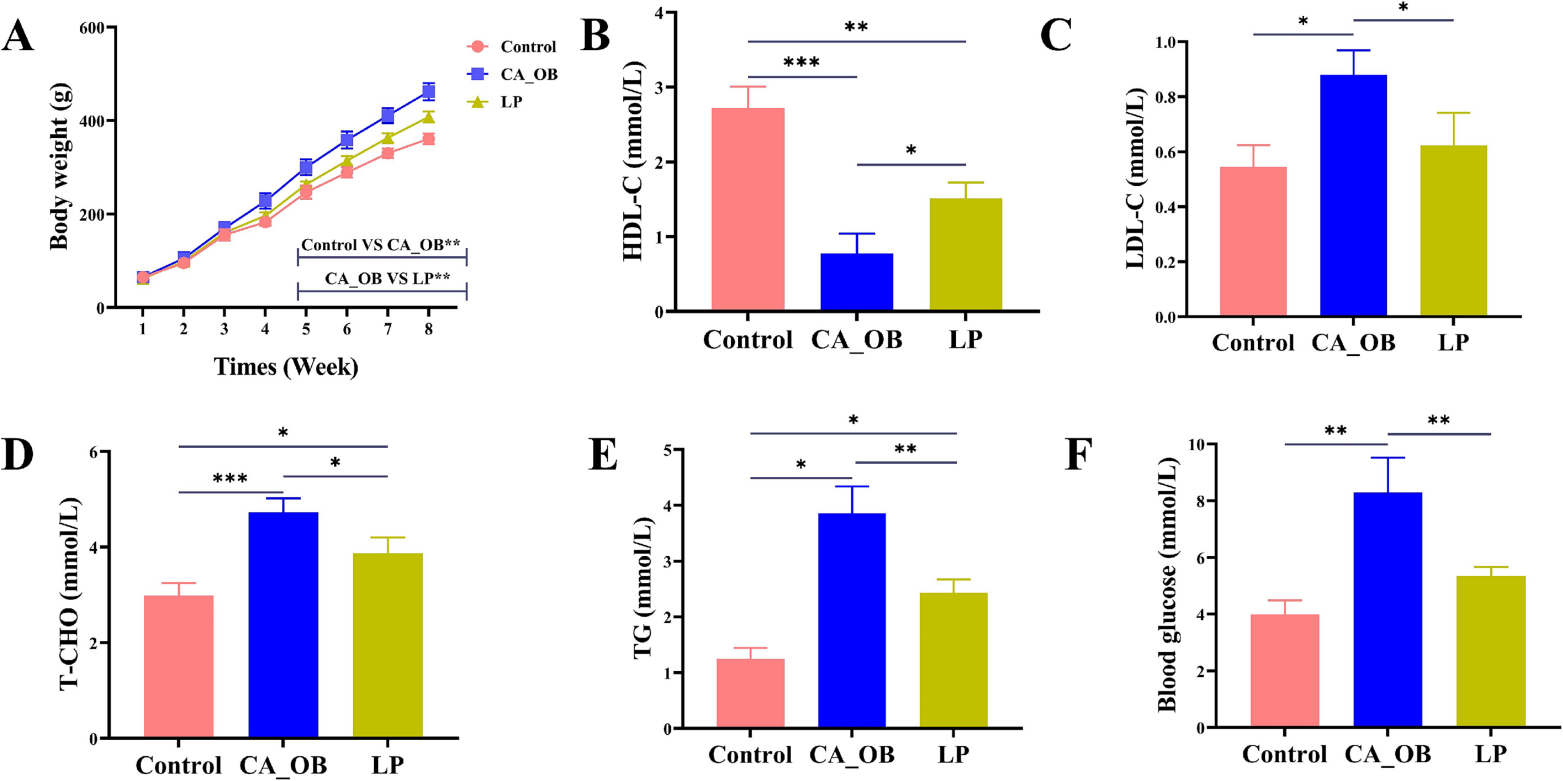
Rat weights and serum indices in relation to *L. plantarum*. **(A)** Change of rat weights within 8 weeks, **(B)** HDL, **(C)** LDL, **(D)** T-CHO, **(E)** TG, and **(F)** Blood glucose. Values are means ± SD. There were three biological duplicates (*n* = 3). * *p* < 0.05, ** *p* < 0.01, *** *p* < 0.001.

After an eight-week feeding period, a clear change in weight was noted among the rats in the LP group, with the CA_OB group displaying a much greater weight than their Control counterparts. Rats within the LP group showed significantly lower weights compared to those in the CA_OB group (Figure 8A). Compared with the Control group, the serum TG, T-CHO, LDL-C and Blood glucose levels in the CA_OB group were significantly increased, while the serum HDL-C level was significantly decreased, indicating that the blood lipid metabolism in CA_OB group was abnormal. However, the LP group significantly inhibited the lipid parameters in rat serum (Figures 8B–F).

#### 3.1.9 H&E staining sections of rat liver and fat

**Figure 9:**
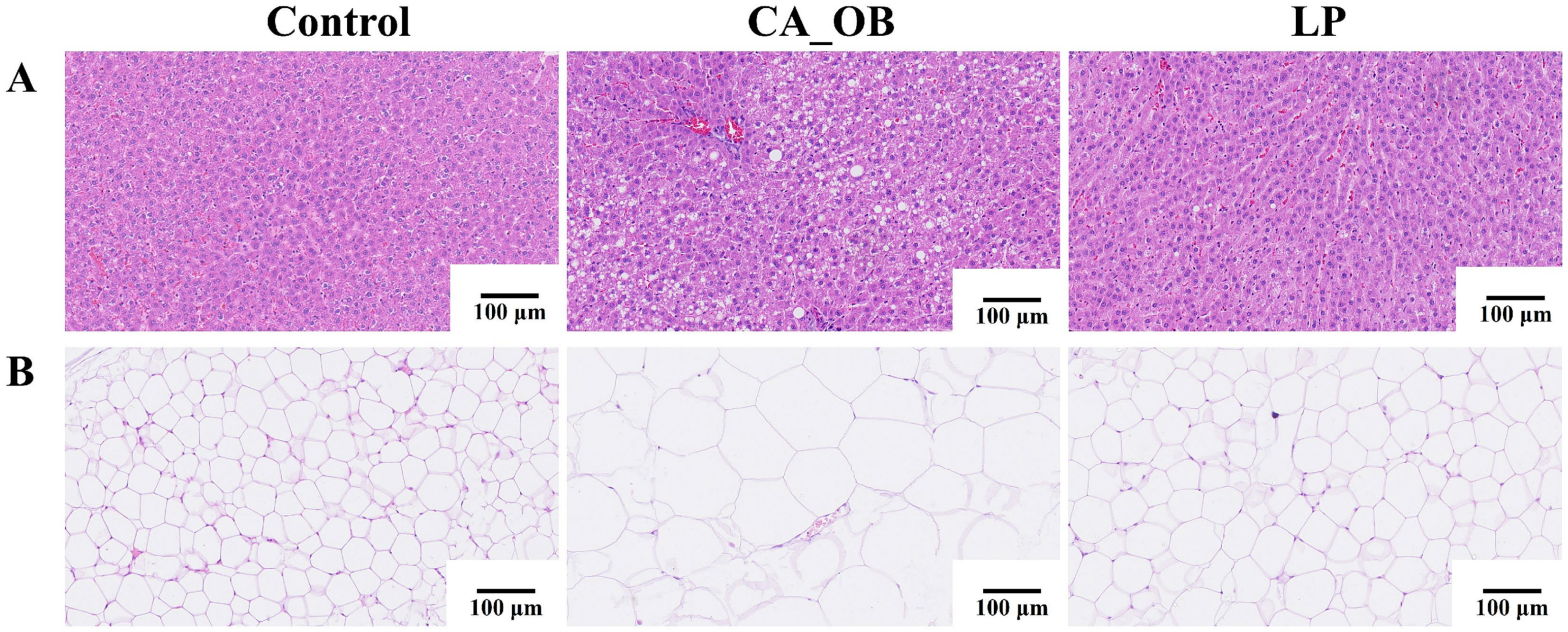
When rats are given *L. plantarum*, it can prevent their blood lipid levels from rising. **(A)** The morphological change of liver tissue in rats by H&E staining, **(B)** the change of epididymal adipose tissue in rats by H&E staining. Data are represented as means ± SD. There were three biological duplicates (*n* = 3).

The results indicated no significant pathological changes in the liver tissue of rats from the Control group. In the CA_OB group, liver cells were obviously swollen, filled with fat vacuoles of different sizes, and inflammatory cells were obviously infiltrated. Rats from the LP group had far fewer fat vacuoles in their liver tissue, although there were still some localized inflammatory reactions (Figure 9A). The CA_OB group’s fat cells were noticeably larger and there were less cells in the same field of vision when compared to the group on a regular diet. Rat epididymal adipocyte hypertrophy may be inhibited following treatment with *L. plantarum*, suggesting that probiotic supplementation has a beneficial impact on lipogenesis (Figure 9B).

#### 3.1.10 16S rRNA gene sequencing of intestinal flora

**Figure 10:**
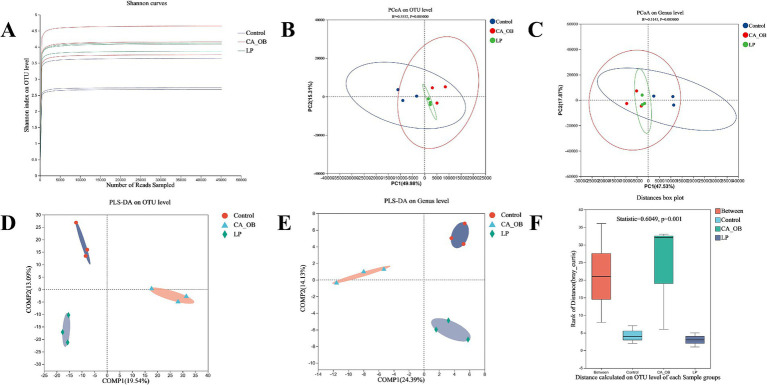
Effect of adding *L. plantarum* on the diversity of intestinal flora. **(A)** Shannon curves, **(B)** PCoA plot of the intestinal flora on OTU level, **(C)** PCoA on Genus level, **(D)** PLS-DA on OTU level, **(E)** PLS-DA on Genus level, **(F)** Beta diversity analysis of ANOSIM analyze on OTU level. There were three biological duplicates (*n* = 3). Principal coordinate analysis (PCoA); Partial least-squares discriminant analysis (PLS-DA); Analysis of similarities (ANOSIM); Operational taxonomic unit (OTU).

To assess the diversity of bacterial communities, the dilution curve was primarily constructed using the Alpha diversity index from samples taken at various sequencing depths. The Shannon index is an important metric that tends to smooth out the dilution curve, serving as a reflection of the diversity present within microbial communities. This index indicates that the data volume gathered from each group is sufficient to meet the sequencing requirements (Figure 10A). Results from the Principal Coordinate Analysis highlighted that the contributions of Principal Component 1 and Principal Component 2 exceeded 60%, allowing for a comprehensive representation of the community composition. The LP group was similar to the Control group at the OTU level, and the CA_OB group and the LP group could be distinguished more clearly (Figure 10B). The PCoA data revealed that the LP group and the Control group nearly completely overlapped at the genus level, further supporting the idea that lactic acid bacteria might govern the gut flora (Figure 10C). To evaluate whether the differences between groups were statistically significant, we employed supervised partial least squares analysis. The findings from this analysis clearly illustrated a notable separation between the groups at both the OTU level and the genus level (Figures 10D,E). The ANOSIM analysis further showed the significant differences between groups (Figure 10F).

**Figure 11:**
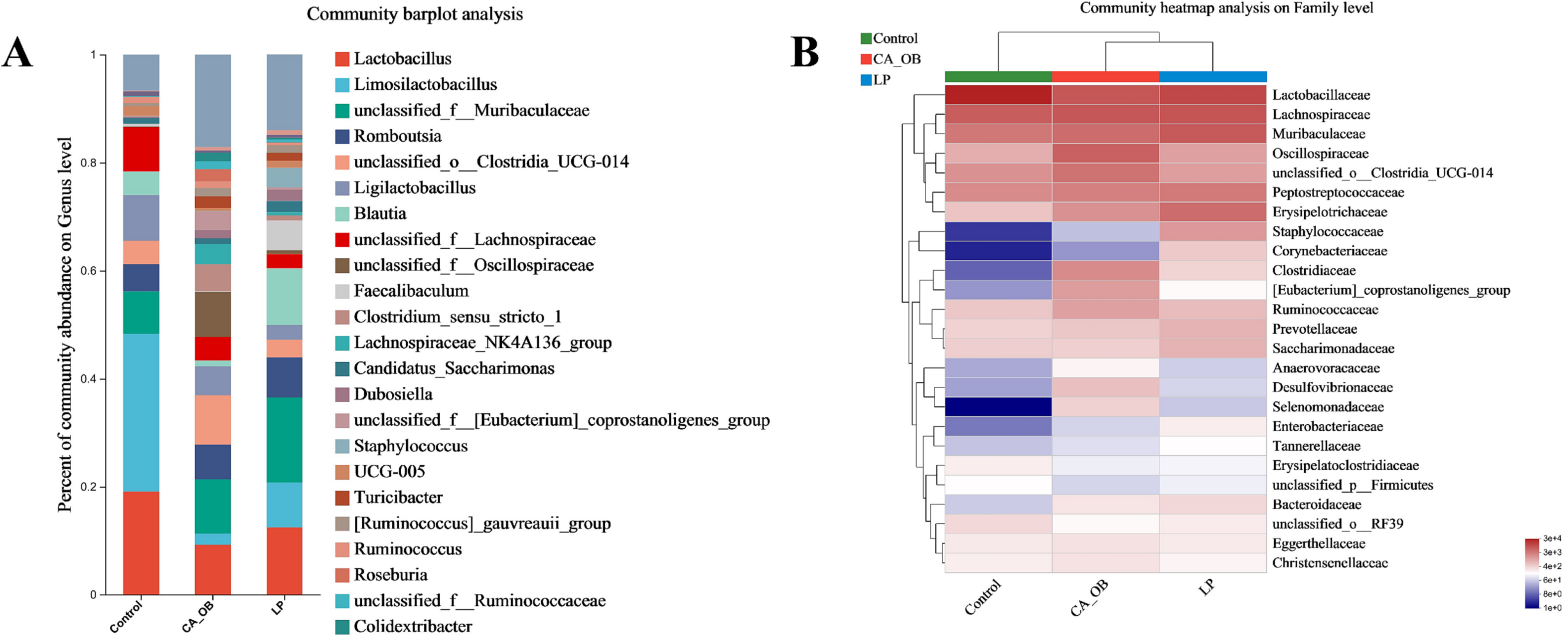
At different classification levels, the species composition and relative abundance of various species were counted. **(A)** Comparative genus-level abundance of the gut flora, **(B)** family-level gut flora’s relative abundance. There were three biological duplicates (*n* = 3).

Intestinal microbiota may shield the host against pathogens that cause intestinal disorders in addition to enhancing nutrition absorption and preserving immunity. On the genus of level, the LP group increased relative abundance of *Lactobacillus*, *Limosilactobacillus*, *unclassified_f__Muribaculaceae*, *Blautia*, *Faecalibaculum*. The LP group led to the decrease of the abundance of pathogenic bacteria, including *unclassified_o__Clostridia_UCG-014*, *unclassified_f__Oscillospiraceae*, *Turicibacter*, *unclassified_f__Lachnospiraceae*, *Clostridium_sensu_stricto_1* (Figure 11A).

On the family of level, the three groups of bacteria were mainly the following: *Lactobacillaceae*, *Lachnospiraceae*, *Muribaculaceae*, *Oscillospiraceae*, *unclassified_o__Clostridia_UCG-014*, *Peptostreptococcaceae*, *Erysipelotrichaceae* (Figure 11B).

**Figure 12:**
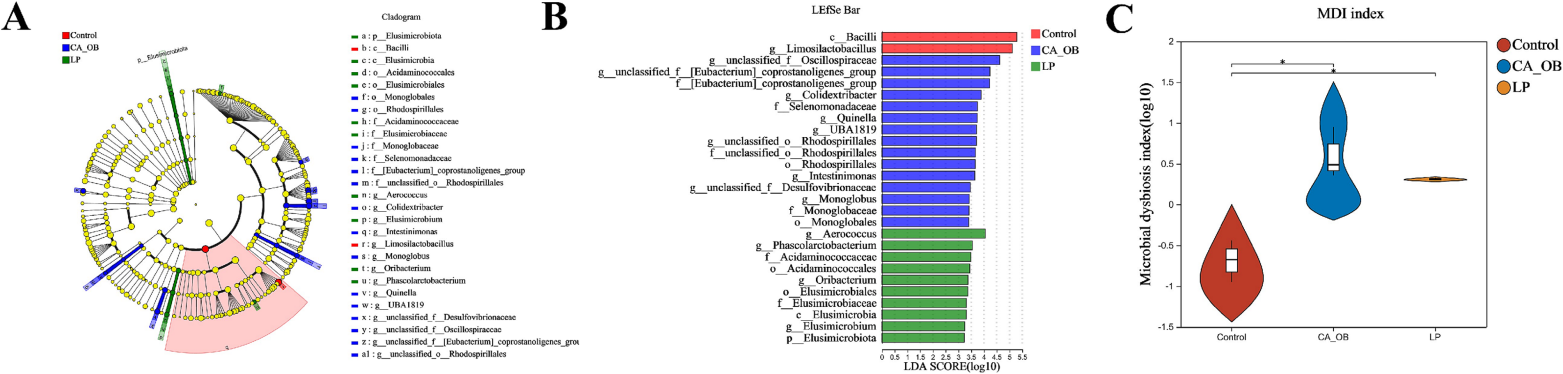
Analysis on interspecific differences of intestinal flora. **(A,B)** The LEfse multi-level species differences was conducted, **(C)** the microbial dysbiosis index in different groups. There were three biological duplicates (*n* = 3). Linear discriminant analysis effect size (LEfse).

This analysis illuminated the communities or species that exhibited significant variation across all taxonomic levels, including phylum, class, order, family, and genus. It was evident that the relative importance of microbial biomarkers escalated in tandem with increasing Linear Discriminant Analysis (LDA) scores. Compared with the Control group, in the CA_OB group, *g__unclassified_f__[Eubacterium]_coprostanoligenes_group*, *f__Selenomonadaceae*, *g__unclassified_f__Oscillospiraceae, g__Quinella, g__unclassified_f__Desulfovibrionaceae*. Contrasting to the CA_OB group, The profusion of *g__Phascolarctobacterium, f__Acidaminococcaceae, g__Elusimicrobium and c__Elusimicrobia* within the LP group was markedly elevated (Figures 12A,B). The degree of microbial ecological imbalance was measured using the microbial dysbiosis index. The degree of microbial imbalance increases with increasing value. In comparison to the Control group, the CA_OB group had a considerably higher microbial dysbiosis index. The microbial dysbiosis index clearly decreased following *L. plantarum* intervention, suggesting that *L. plantarum* may control the intestinal flora of CA_OB rats (Figure 12C).

Phylogenetic Investigation of Communities by Reconstruction of Unobserved States (PICRUSt2), Kyoto Encyclopedia of Genes and Genomes (KEGG), Evolutionary Genealogy of Genes: Non-supervised Orthologous Groups (EggNOG).

**Figure 13:**
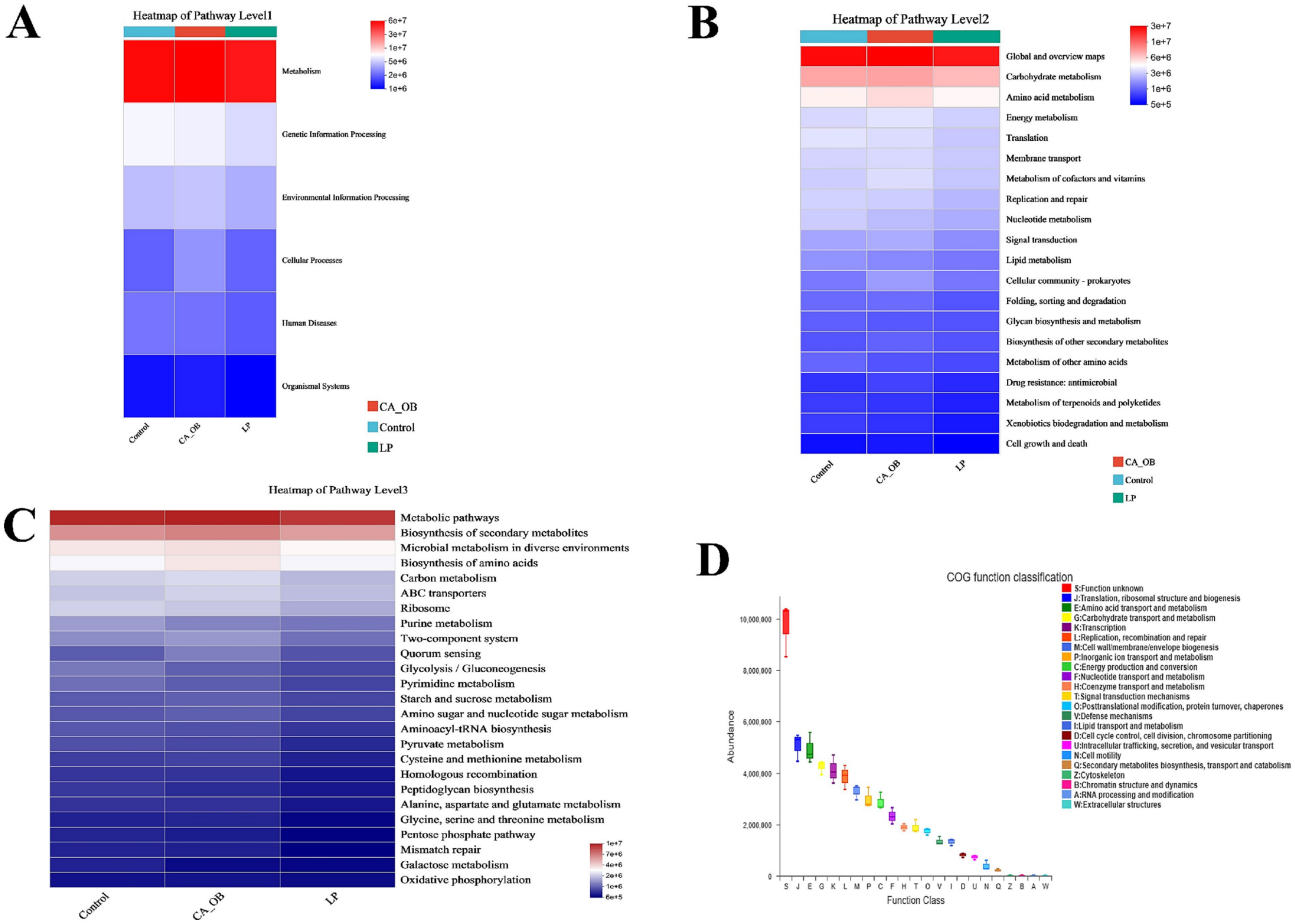
Prediction and analysis of intestinal flora function. **(A–C)** The function of PICRUSt2 in conjunction with the KEGG database to anticipate intestinal microorganisms showing result of the KEGG pathway at Level l **(A)**, Level 2 **(B)** and Level 3 **(C)**, **(D)** PICRUSt2 is combined with the EggNOG database to forecast the function of intestinal microorganisms. There were three biological duplicates (*n* = 3).

Bacterial colony function is largely associated with the metabolism pathway, according to the 16S rRNA sequencing data and KEGG functional predictions (Figure 13A). We could further demonstrate that bacteria are related to global and overview maps, carbohydrate metabolism, and amino acid metabolism by combining 16S rRNA sequencing data with KEGG data information of Pathway Level 2 (Figure 13B). Based on the 16S rRNA data and the PICRUSt2 channel display of EggNOG and KEGG databases, we predicted the biological functions of bacteria. 16S rRNA sequencing data combined with KEGG data information of Pathway Level 3, we could further confirm that bacteria are related to purine metabolism, peptidoglycan biosynthesis, oxidative phosphorylation, amino acid metabolism, streptomycin biosynthesis, nitrogen metabolism, taurine and hypotaurine metabolism (Figure 13C). In an effort to delve deeper into the functional roles of bacteria, we utilized the EggNOG database. Predictions indicated that the primary functions of the bacteria were closely associated with metabolic processes. These processes predominantly involved translation, the structure and biogenesis of ribosomes, as well as the transport and metabolism of amino acids and carbohydrates (Figure 13D).

**Figure 14:**
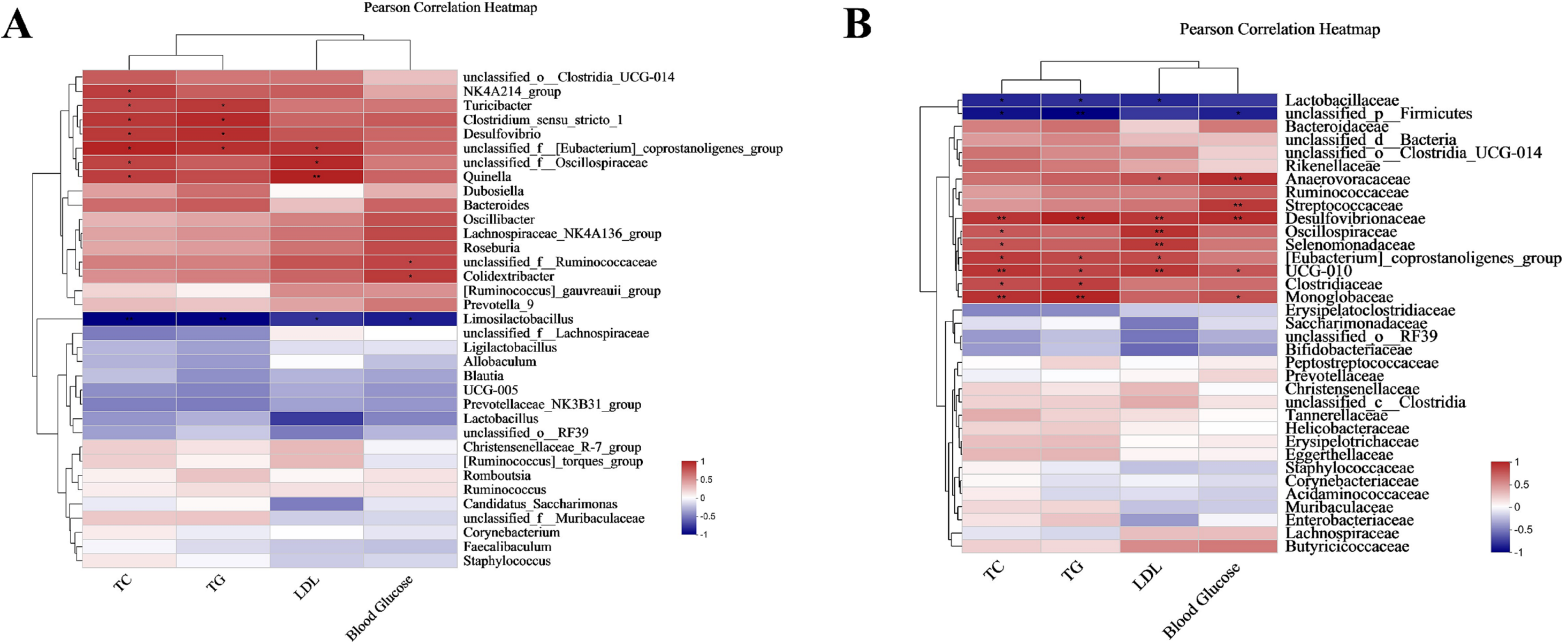
Pearson correlation between lipid metabolism index and microbiota. **(A)** Pearson correlation between the genus and lipid metabolism index. **(B)** Pearson correlation between the family and lipid metabolism index. Correlations were represented with a red color indicating positive relationships and blue indicating negative ones. There were three biological duplicates (*n* = 3). * *p* < 0.05, ** *p* < 0.01, *** *p* < 0.001.

At the genus level, *Limosilactobacillus* was negatively connected with associated with obesity variables (TC, TG, LDL, Blood Glucose), while *Clostridium_sensu_stricto_1*, *Turicibacter*, *unclassified_f__Oscillos*p*iraceae*, *Colidextribacter*, *Quinella*, *Desulfovibrio* were positively linked to some relating to obesity factors (Figure 14A). At the family level, *Lactobacillaceae*, *unclassified_p__Firmicutes* had a negative correlation with linked to obesity indexes, while *Streptococcaceae*, *Desulfovibrionaceae*, *Clostridiaceae*, *Oscillospiraceae*, *Selenomonadaceae*, *UCG-010* were found to have a positive correlation with obesity measures (Figure 14B).

## 4 Discussion

There is an urgent need for novel approaches to the prevention and management of dental caries and obesity, two disorders that have a negative impact on one’s physical and well-being due to their rising prevalence. One promising candidate is *L. plantarum*, a genus of *Lactobacillus* that has recently been reclassified and is noted for its extensive metabolic diversity (31, 32). Prior studies showed that *L. plantarum* ATCC14917 inhibited the growth of *S. mutans*, which causes dental caries, and broke down the structure of harmful biofilm (33). Research findings suggest that incorporating *L. plantarum* into health regimens may help mitigate the onset of conditions such as atherosclerosis and non-alcoholic fatty liver disease (34, 35). However, it was little known whether *Lactobacillus* will affect the treatment of clinical diseases with dental caries and obesity.

Biofilm was formed by microbial communities to withstand different environments and shield bacterial cells with tenacious adherence (36). The results showed that the yield was the lowest at 0 h, and the regulation effect on biofilm formation of *S. mutans* was better at 12 h, and early intervention had a better influence on biofilm formation. It was speculated that the antibacterial products in the supernatant of *L. plantarum* played a role and affected the initial adhesion and colonization of pathogenic bacteria. In the later stage of biofilm formation, *L. plantarum* supernate was still effective on the original mature *S. mutans* biofilm, which was similar to the previously reported results (37). A number of *in vitro* parameters, such as continuous human digestion, gastric juice and bile production, and pathogen resistance, can be used to assess the health advantages of *Lactobacillus* (38). *L. plantarum* has a high viable count in intestinal and simulated artificial gastric juice, which is in line with other study findings and suggests that the strain may withstand the hostile conditions of the gastrointestinal tract and thrive (39).

Probiotics’ ability to self-aggregate is typically what allows them to adhere to mucous membranes and epithelial cells’ surfaces (40). According to the current study, *L. plantarum* ATCC 14917’s capacity for self-aggregation grew over time. Through the processes of saliva ingestion, *L. plantarum* forms co-aggregates with pathogenic bacteria. This process effectively reduces the pathogenic bacteria that float within the oral cavity, thereby minimizing the potential for these harmful microorganisms to colonize the oral environment (41). The experimental results show that the strains to be tested have a higher copolymerization ability in the early stage (4 h) and a lower copolymerization ability in the late stage (24 h), which may be because the strains and determination times differ.

Dental caries was a typical biofilm-induced disease (42). Microorganisms present in the oral cavity are a crucial factor in the development of dental caries; however, they alone are insufficient, as the creation of cariogenic biofilm relies on the host’s dietary habits (43). It was noted that unlike *S. mutans*, none of the tested strains of Lactobacillus demonstrated a meaningful increase in biofilm formation and did not produce water-insoluble sticky extracellular polysaccharides under similar growth conditions when cultured in sucrose-enriched medium compared to when they were grown in a medium lacking sucrose (44, 45). Thus, we hypothesize that it might be because *S. mutans* continuously produces sugar using sucrose as a substrate. Conversely, *L. plantarum*, categorized as a heterotrophic bacterium, is limited to consuming carbohydrates and not able to make sugar, aligning with the *in vitro* experimental findings that showcased its anti-caries properties.

The oral cavity is an important passage into the human body (46). The first stage of digestion involves the mechanical breakdown of food by the teeth, which, in conjunction with saliva, forms a cohesive mass that subsequently progresses into the gastrointestinal tract (47). Thus, it seemed sense that probiotics would affect the microbial flora of the mouth cavity in the same way as they affect the intestinal system (48). It has been revealed that among the overweight population, the number of Firmicutes increased while the number of Bacteroides declined (49). *S. mutans* belongs to Firmicutes (50). The formation of fats is directly linked to the fermentation and absorption of polysaccharides produced by gut microorganisms through a balanced diet (51). Consequently, we conjectured that *S. mutans* would promote fat accumulation while *L. plantarum* might inhibit it. These findings suggest that the *Lactobacillus* may prevent obesity and tooth cavities from developing.

By creating an animal model, the function of *L. plantarum* ATCC 14917 *in vivo* was further confirmed. With particular emphasis on the density of hard tissue, Micro-CT was suggested as a trustworthy technique for determining the volume of tooth hard tissue (52). Consequently, the mandibular enamel volume was computed in this study. The results show that *L. plantarum* played a significant anti-caries role.

Furthermore, prolonged consumption of high-fat and high-sugar diets can significantly alter the diversity and composition of microbial communities within the gut, as well as modify the intestinal environment (53). Probiotics have the potential to directly alleviate diseases by modulating the ratio of good to harmful microorganisms within the microbial flora (54). *Unclassified_f__Muribaculaceae* belongs to Bacteroides S24-7, which usually exists in the intestinal microbiota of healthy mice (55). The findings of this study indicate that gymnosperms classified within the CA_OB group exhibited a lower abundance compared to those in the LP group. Moreover, the *unclassified_f__Muribaculaceae* was shown to play a significant role in regulating body weight, as well as in the degradation of food items and polysaccharides (56). This family is also responsible for the production of short-chain fatty acids (SCFAs), which include important metabolites such as succinic acid and propionic acid (57). In the context of clinical research, *Lactobacillus* and *Bifidobacterium* are among the most frequently employed probiotic strains. *Lactobacillus* can alter the synthesis pathway of lipids and carbohydrates, convert sugar to lactic acid (58).

Both *unclassified_f__Ruminococcaceae* and *Ligilactobacillus* have beneficial effects on the composition of intestinal microbiota, preserving the steady state of the intestinal milieu and emerging as important elements of the microbiota of healthy individuals (59, 60). *Unclassified_f__Ruminococcaceae* is one of the most effective bacterial genera for decomposing carbohydrates (61). It is a member of the *Ruminococcaceae* family of bacteria, multiple of which are known to make butyrate (62). SCFAs was found to be produced by the *unclassified_f__[Eubacterium]_coprostanoligenes_group* (63). It is positively correlated with fecal butyrate content. On a high-fat diet, it has been found that *unclassified_f__[Eubacterium]_coprostanoligenes_group* have a lipid-lowering impact (64). *Limosilactobacillus* can help with weight management, liver disease relief, increasing intestinal integrity, immunological regulation, and improving glucose homeostasis (65). Another lactic acid-producing bacterium, *Escherichia coli*, was found in the fecal samples, showing no significant difference across other groups. Its presence has been associated with anti-inflammatory properties and the enhancement of the intestinal barrier (66). The phylum Firmicutes includes the genus *Blautia*, which is recognized for its potential in mitigating intestinal inflammation (67). This inflammatory response has been linked to the regulation of G-protein coupled receptors, specifically GPR41 and GPR43 (68).

In the CA_OB group, the abundance of *unclassified_o__Clostridia_UCG-014* and *unclassified_f__Oscillospiraceae* increased, and this was positively connected with serum indices. A conditional pathogen linked to numerous illnesses is *unclassified_o__Clostridia_UCG-014* (69). Furthermore, it is believed that *unclassified_f__Oscillospiraceae* is connected to liver disorders (70) and damage (71). The CA_OB group exhibited a significantly higher abundance of *Clostridium_sensu_stricto_1* and *Turicibacte* in comparison to the LP group. These genera contain significant pathogenic bacteria that, through causing toxins that lead to intestinal infections and encouraging chronic inflammation, contribute to the obesity process (72, 73). A type of bacteria found in the digestive tract called *unclassified_f__Lachnospiraceae* is linked to an increase in lipid levels and fat mass (74). Glucose and lipid metabolism are positively correlated with *unclassified_f__Lachnospiraceae* (75). *Desulfovibrionaceae* is a type of sulfate-reducing bacteria that produces more H_2_S (76). Inflammation results from H_2_S’s reduction of disulfide bonds in the mucus network, which breaks the mucus barrier and exposes epithelial cells to toxins and germs (77). According to some research, a probiotic-rich diet can reduce the quantity of *Desulfovibrionaceae* in obese people (78). The above results showed that *L. plantarum* could adjust the richness of other flora and the composition of intestinal flora, and play different functions.

## 5 Conclusion

This investigation also delved into the impacts of *L. plantarum* on both intestinal and oral health. The results demonstrated impressive antibacterial and anti-obesity effects of *L. plantarum* against the oral pathogen *S. mutans in vitro*. Through the use of Micro-CT imaging, the study confirmed a significant disparity in the incidence of dental caries between the LP and CA_OB groups. Furthermore, its strain improved fat accumulation in rat liver and markedly regulated the abnormality of lipid metabolism. Consequently, it was anticipated that *L. plantarum* ATCC 14917 would find applications in the medical and healthcare fields to help maintain the integrity of the intestinal barrier and the equilibrium of intestinal flora.

## Data Availability

The datasets presented in this article are not readily available because due to laboratory policies and confidentiality agreements, raw data cannot be provided. Requests to access the datasets should be directed to Xiaopeng Yang, 82910923@qq.com.
